# Disodium citrate-assisted hydrothermal synthesis of V_2_O_5_ nanowires for high performance supercapacitors

**DOI:** 10.1039/c7ra12607g

**Published:** 2018-01-16

**Authors:** Shanshan Pan, Ling Chen, Yahao Li, Shuolin Han, Lin Wang, Guangjie Shao

**Affiliations:** Hebei Key Laboratory of Applied Chemistry, College of Environmental and Chemical Engineering, Yanshan University Qinhuangdao 066004 China; State Key Laboratory of Metastable Materials Science and Technology, Yanshan University Qinhuangdao 066004 China

## Abstract

Orthorhombic vanadium pentoxide (V_2_O_5_) nanowires with uniform morphology were successfully fabricated *via* a facile hydrothermal process. The effect of disodium citrate dosage on the crystallinity, morphology and electrochemical properties of the products was analyzed. Experimental results indicate that orthorhombic V_2_O_5_ nanowires with high crystallinity and diameter of about 20 nm can be obtained at 180 °C for 24 h when the dosage of disodium citrate is 0.236 g. Furthermore, the prepared V_2_O_5_ nanowires demonstrate a high specific capacitance of 528.2 F g^−1^ at 0.5 A g^−1^ and capacitance retention of 85% after 1000 galvanostatic charge/discharge cycles at 1 A g^−1^ when used as supercapacitors electrode in 0.5 M K_2_SO_4_.

## Abstract 2

Orthorhombic vanadium pentoxide (V_2_O_5_) nanowires with uniform morphology were successfully fabricated *via* a facile hydrothermal process.

## Graphical abstract

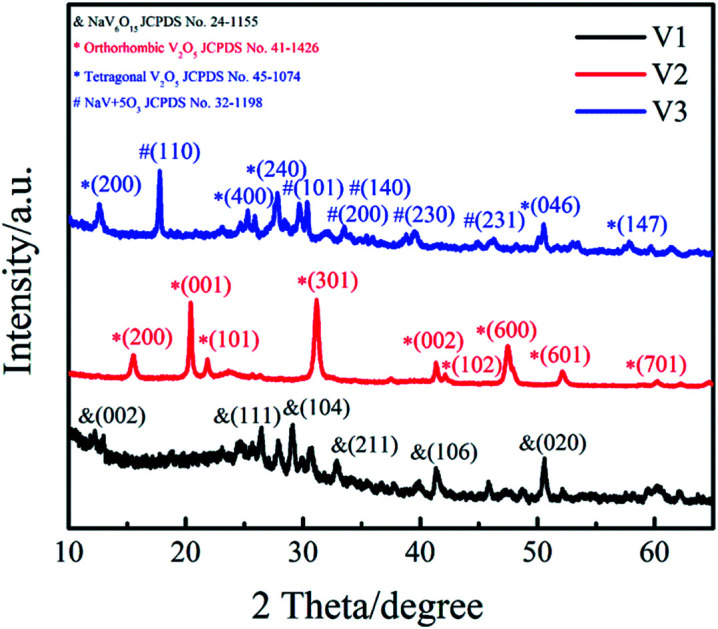

## 1. Introduction

Supercapacitors are considered to be one of the most promising energy storage devices due to their higher energy density compared to conventional capacitors, higher power density than batteries, long cycle life, and environmental friendliness.^1–8^ As electrode materials for supercapacitors, vanadium pentoxide (V_2_O_5_) has attracted much attention because of its extensive properties such as layered structure, low cost, high theoretical capacity, as well as stable working voltage.^9–14^ However, the collapse of the layered structure due to insertion/extraction of ions greatly limits its application in supercapacitors.^14^ In order to overcome the above shortcomings, effective methods have been devoted to synthesize nanostructured V_2_O_5_, which is beneficial to shorten diffusion path for ions, enhance contact area between electrode and electrolyte, and improve capacitive property.^14–16^

Recently, many researchers have studied to synthesize diverse structures of V_2_O_5_ nanomaterials, such as nanowires,^14,17–21^ nanoblets,^22^ nanotubes,^23^ nanoflowers,^20^ nanoballs^24–26^ and so on. Various synthetic methods including sol–gel route,^25^ flame spray method,^27^ hydrothermal and solvothermal method for different structures of V_2_O_5_ have been reported.^17–20^ Among them, the hydrothermal and solvothermal method is considered to be a convenient and versatile approach which possesses potential advantages of high crystallinity and purity, as well as well-controlled morphology. Wang *et al.*^18^ synthesized V_2_O_5_ nanowires using [VO(O_2_)_2_(OH_2_)]^−^ as the starting material through a hydrothermal route for 96 h, which exhibited a specific capacitance of 351 F g^−1^ when used as supercapacitor electrode in 1 M LiNO_3_. The preparation of uniform V_2_O_5_ nanoballs displaying a specific capacitance of 479 F g^−1^ by a solvothermal treatment of ammonium metavanadate at 180 °C for 48 h has been reported by Yang *et al.*^9^ Orthorhombic single-crystalline V_2_O_5_ nanobelts with 30–80 nm in width and 30–40 nm in thickness were synthesized through a hydrothermal process at 180 °C for 24 h but the electrochemical properties weren't explored.^10^ Nevertheless, there are some problems such as low solubility of raw material, easy agglomeration of final products and long reaction time for hydrothermal and solvothermal methods. As is known, the way to overcome these limitations by additive agent has drawn great interest of researchers.^22–24^ However, there are few reports on preparation of V_2_O_5_ nanomaterials using disodium citrate as additive agent. Saravanakumar *et al.*^28^ constructed V_2_O_5_ nanoporous network *via* simple capping agent-assisted precipitation technique with disodium citrate as capping agent, showing the specific capacitance of 316 F g^−1^ with 0.5 M K_2_SO_4_ electrolyte.

Herein, we reported a facile and efficient capping agent-assisted hydrothermal method to prepare uniform orthorhombic V_2_O_5_ nanowires with good crystallinity and about 20 nm in diameter. The results illustrate that the phase and morphology of the products are sensitive to the amount of disodium citrate in the synthesis process. Moreover, a high specific capacitance of 528.2 F g^−1^ for the as-prepared V_2_O_5_ nanowires has been achieved when used as a supercapacitor electrode in 0.5 M K_2_SO_4_. In addition, providing V_2_O_5_ nanowires about 20 nm in diameter within short reaction time, this synthesis method used in this article shows great application prospects.

## 2. Experimental

### 2.1. Preparations of V_2_O_5_ nanowires

All the reagents used in experiments were of analytical grade and used without further purification. V_2_O_5_ nanowires were synthesized by a facile capping agent-assisted hydrothermal process with disodium citrate as capping agent. In a typical synthesis procedure, 0.364 g of V_2_O_5_ was dissolved in 30 mL of distilled water, followed by slow dropping of 15 mL of 30% H_2_O_2_ solution at a rate of 15 mL min^−1^ with magnetic stirring. The yellow suspension gradually turned into orange solution with the addition of H_2_O_2_, which is stirred for 2 h to form a transparent ruby red color solution. Then, disodium citrate of 0.118 g, 0.236 g and 0.472 g were added to the solution, respectively. Afterwards, the mixture was transferred into a 100 mL Teflon-lined stainless steel autoclave and kept at 180 °C for 24 h. Finally, the solution was cooled to room temperature naturally, washed by alcohol in a centrifuge and dried in a blast oven at 60 °C to obtain V_2_O_5_ precursor. The obtained V_2_O_5_ precursor was placed in a muffle furnace and sintered in air at 300 °C for 2 h to acquire final product. The three final products prepared with disodium citrate in amount of 0.118, 0.236 and 0.472 g were labeled as V1, V2 and V3, respectively.

### 2.2. Characterization

The phase of the prepared samples were identified by X-ray diffraction (XRD) on a Rigaku Smart Lab X-ray diffractometer operated at 40 kV using Cu Kα radiation at a scan rate of 5° min^−1^. The morphologies of the samples were observed by transmission electron microscopy (TEM, Hitachi HT 7700) and scanning electron microscopy (SEM, Hitachi SUPRA-55).

### 2.3. Electrochemical measurements

Electrochemical performances of the supercapacitor cells were tested by Cyclic Voltammetry (CV), galvanostatic charge/discharge (GCD, Neware battery testing system, Shenzhen Neware instrument, China). Electrochemical impedance spectroscopy (EIS, CHI660e electrochemical workstation, Shanghai CH instrument, China). All tests were carried out in a three-electrode system, in which Pt and Hg/Hg_2_SO_4_ electrode were used as the counter and the reference electrode, respectively. The working electrodes were comprised of 80 wt% of active material, 15 wt% of carbon black and 5 wt% of polytetrafluoroethylene (PTFE) binder on nickel grid. The mass loading of the active materials in the electrodes is about 2 mg cm^−2^. The electrolyte was 0.5 M K_2_SO_4_. CV data were collected between −0.4 to 0.8 V at a scan rate of 5 mV s^−1^ and GCD tests were performed in the potential range of −0.1 to 0.6 V. The potential window is a little narrow in comparison to that in CV method, which is a optimized results. EIS tests were carried out in the frequency range of 0.01–10^5^ Hz at a 5 mV amplitude referring to open circuit potential. The mass specific capacitances were calculated by using the following equations.^5^

1 *C* = (*I*Δ*t*)/(*m*Δ*V*)

where *I* (A) is the constant discharge current, Δ*t* (s) is the time needed for discharge, *m* (g) is the mass of the active material in the working electrode, Δ*V* (V) is the discharge potential.

The cyclic stability of V_2_O_5_ nanowires V2 were also measured at 1 A g^−1^ for 1000 GCD cycles.

## 3. Results and discussion

**Fig. 1:**
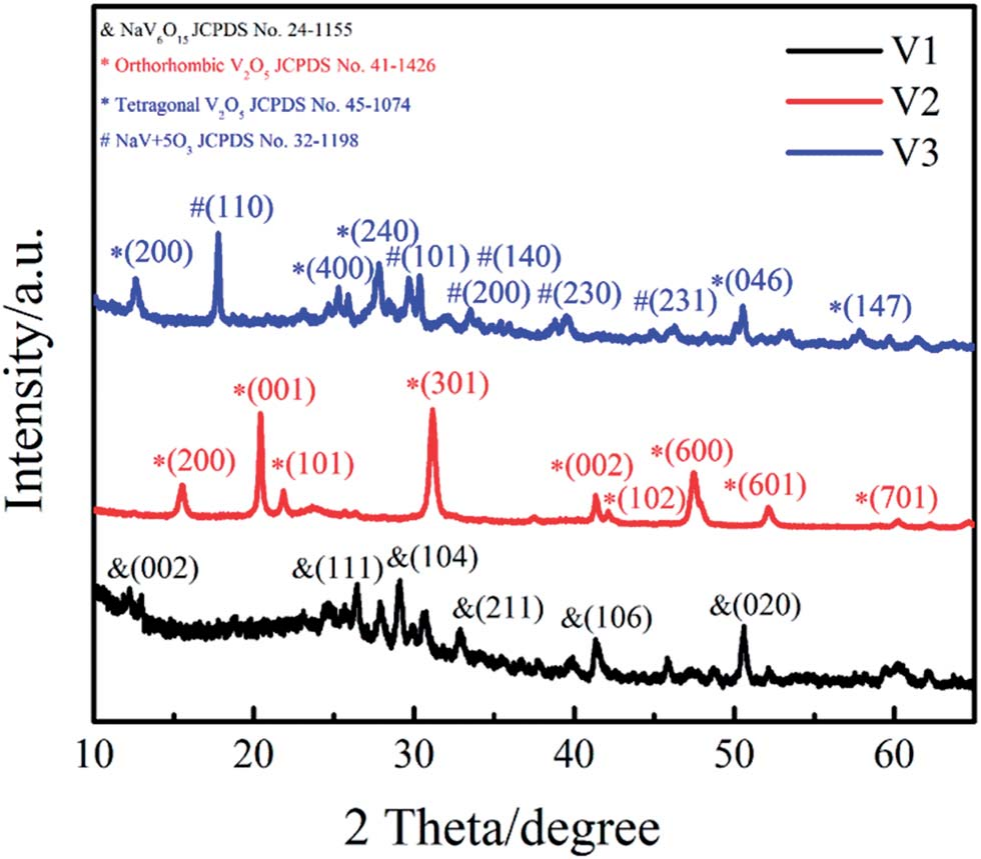
XRD patterns of samples (V1, V2 and V3).

Phase structure of the samples (V1, V2, V3) were determined by XRD and patterns are presented in Fig. 1. It can be clearly observed that the featured diffraction peaks of V2 at 2*θ* of 15.5°, 20.4°, 21.8°, 26.3° and 31.1° corresponding to the (200), (001), (101), (110) and (301) planes. The marked diffraction peaks indicate that the XRD spectrum of V2 agrees well with orthorhombic V_2_O_5_ (JCPDS 41-1426; space group: *pmmn* (59)). The sample V1 was obtained by reducing the amount of disodium citrate, its characteristic diffraction peaks at 2*θ* of 26.5°, 29.3° and 50.7° are correspond to the (111), (104) and (020) planes, matching well with NaV_6_O_15_ (JCPDS 24-1155). The XRD spectrum of V3 shows that different diffraction peaks at 2*θ* = 12.4°, 24.9° and 50.6° can be indexed to the (200), (400) and (046) planes of cubic V_2_O_5_ (JCPDS 45-1074), and the peaks at 17.7°, 29.6° and 30.2° are corresponded to the (110), (101) and (140) planes of NaV+5O_3_ (JCPDS 32-1198). The results illustrate the sample V3 is a mixture of cubic V_2_O_5_ and NaV+5O_3_. These results suggest that the amount of disodium citrate plays an important role in controlling the crystal phase of the products. Moreover, it can be found that the sample V2 shows higher degree of crystallinity and purity than V1 and V3.

**Fig. 2:**
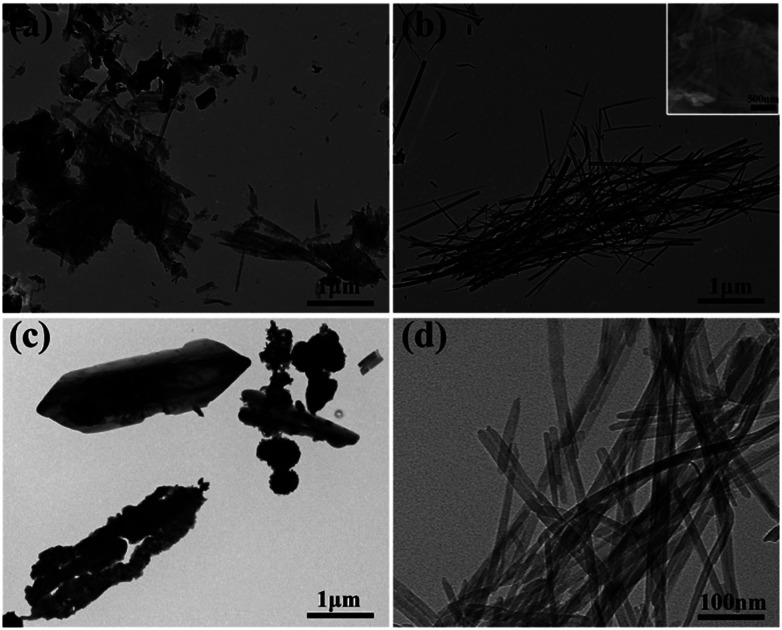
TEM images of the prepared V_2_O_5_ (a) V1, (b) V2, (c) V3 and (d) V2; the inset in panel (b) show the SEM image of V2.

The morphologies of the as-prepared samples (V1, V2, V3) were measured by TEM and SEM (Fig. 2). It can be seen in Fig. 2(a) that the sample V1 is composed of a large quantity of nanosheets and a small amount of nanowires. When the amount of disodium citrate increases to 0.236 g with other experimental conditions unchanged, large amounts of V_2_O_5_ nanowires with a diameter of about 20 nm were obtained for V2 as shown in Fig. 2(b) and (d). Continue to increase the amount of disodium citrate to 0.472 g, the morphology has greatly changed from wires to shuttle type (Fig. 2(c)). As a result, the amount of disodium citrate plays an important role in controlling the morphology of the products.

Besides changing the pH value of hydrothermal solution, the addition of disodium citrate can not only provide Na^+^ for the products such as NaV_6_O_15_ in V1 shown in Fig. 1 but also induce the preferred orientation through selective adsorption on crystal planes.^28^ In addition, disodium citrate is regarded as capping agent to inhibit the agglomeration of nano structures.^28,29^ To sum up, probably under the comprehensive influence of the four factors above, the dosage of disodium citrate obviously affects the phase structure and morphology of the hydrothermal products.

**Fig. 3:**
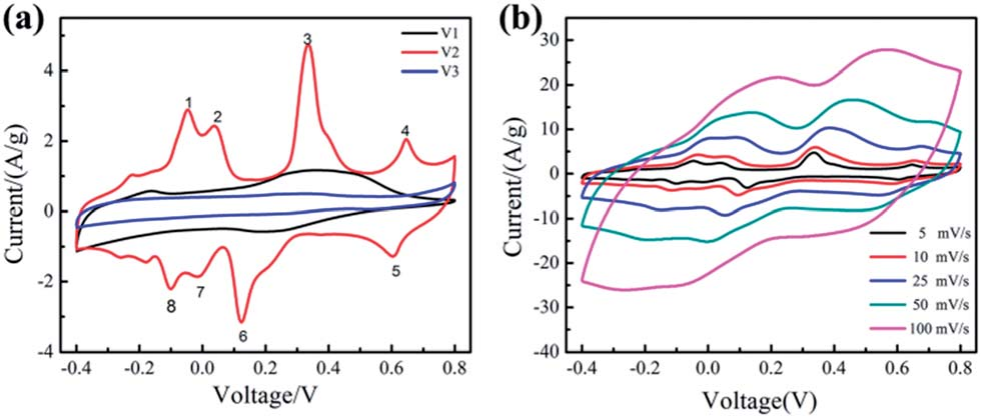
CV curves (a) for V1, V2, V3 at 5 mV s^−1^ and (b) for V2 at 5–100 mV s^−1^.

The electrochemical performances of the samples (V1, V2, V3) were studied using a three-electrode system with 0.5 M K_2_SO_4_ as the electrolyte. Fig. 3(a) shows the CV curves of the samples at 5 mV s^−1^. We can clearly see that, when the amount of disodium citrate is relatively small, the electrode possesses double-layer capacitance and a pair of redox peaks, exhibiting a little pseudocapacitance. With the amount of disodium citrate increases, the CV curve of sample V2 displays four pairs of redox peaks. Similar CV curves with four pairs of peaks can be found for orthorhombic V_2_O_5_ powder in organic electrolyte because of the intercalation/deintercalation of Li^+^ ion^30^ and for hydrated V_2_O_5_ nanowires with layered structure in K_2_SO_4_ aqueous solution due to the insertion/extraction of K^+^ ion.^31^ Such shape with four pairs of peaks is absent in both the CV curve in LiNO_3_ for orthorhombic V_2_O_5_ nanowires prepared by hydrothermal route without the addition of organic surfactant^18^ and the CV curve in K_2_SO_4_ aqueous solution for orthorhombic V_2_O_5_ nanoporous network prepared by hydrothermal route with the addition of disodium citrate.^18^ It seems that the necessary condition for the CV curve with four pairs of peaks needs to be studied furtherly.

The CV results reveal for the first time that the as-prepared orthorhombic V_2_O_5_ nanowires represents obvious pseudocapacitance characteristics derived from the insertion/extraction of K^+^ ion. The electrochemical reaction can be expressed as follows.^18^2
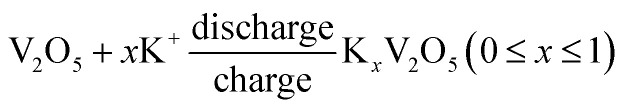


When the amount of disodium citrate is 0.472 g, almost no pseudocapacitance property was detected in the CV curve of V3. The nearly ideal rectangular shape of the CV curves of V3 implies electric double layer capacitance.^32^

The specific capacitance of the samples (V1, V2, V3) based on CV measurement are 263.1, 528.2 and 137.7 F g^−1^, respectively. Obviously, the synthesized orthorhombic nanowires (V2) with uniform morphologies possess the highest specific capacitance, including both double layer capacitance and pseudocapacitance. Its high pseudocapacitance comes from the intercalation/deintercalation of K^+^ ion in its orthorhombic lattice and its high electric double layer capacitance comes from its high surface provided by the uniform nanowires with a diameter of about 20 nm.

Fig. 3(b) displays the CV curves of the sample V2 at different scan rates from 5 to 100 mV s^−1^. The shape of CV curves remain unchanged with the increase of the scan rate, illustrating that the sample V2 exhibits excellent reversibility.^33,34^ Although the area of the CV curve expands with the increasing of the scan rate, a decrease appears in the specific capacitance from 528.2 at 5 mV s^−1^ to 343.0 F g^−1^ at 100 mV s^−1^. This decreasing tendency of specific capacitance can be attributed to the low utilization of active materials. When the scan rate is 5 mV s^−1^, the electrolyte ions can diffuse into small channels. Therefore, the active material is fully utilized and the specific capacitance is higher. The capacitance retention of V2 from 5 to 100 mV s^−1^ is 65%, indicating that the prepared V2 with uniform morphology shows better rate performance.

**Fig. 4:**
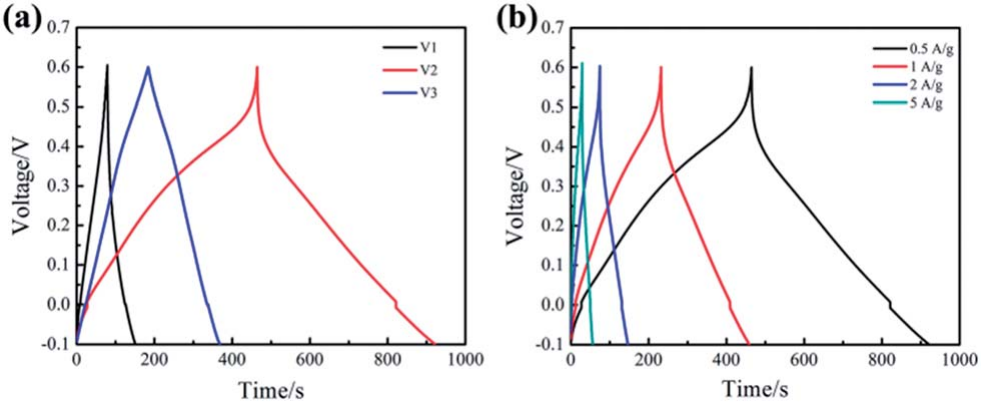
GCD curves (a) for V1, V2, V3 at 0.5 A g^−1^ and (b) for V2 at 0.5–5A g^−1^.

Fig. 4(a) represents the galvanostatic charge/discharge (GCD) curves of the samples (V1, V2, V3) at the current density of 0.5 A g^−1^. Slight curvature in GCD curve of V1 demonstrates that the electrode exhibits most of electrical double layer capacitance characteristics along with a little redox reactions. It is obvious that the GCD curve of V2 is far from linear isosceles triangle, which illustrates the capacitance is mainly contributed by pseudocapacitance. Interestingly, basically isosceles triangle can be observed in the GCD curve of V3, indicating there is almost no pseudocapacitance. The specific capacitance of the samples has been calculated according to the eqn (1) based on the discharge curve.^33^ The specific capacitance of V1, V2 and V3 are 263.1, 528.2 and 137.7 F g^−1^, respectively. These results indicate that the sample V2 possesses the highest specific capacitance, which is consistent with the CV analysis.

Fig. 4(b) shows the GCD curves of V2 at different current densities from 0.5 to 5 A g^−1^. It can be observed that the shape of the GCD curve remains basically unchanged as the current density increasing, which indicates that the prepared V_2_O_5_ nanowires exhibit good rate performance at large current density. In addition, the GCD curve is not an isosceles triangle, illustrating significant the Faraday reaction occurs during charge–discharge process. The sample V2 exhibits a specific capacitance of 357.2 F g^−1^ at 5 A g^−1^. The specific capacitance retention is 68% from 0.5 to 5 A g^−1^, which is close to the results calculated from the CV curves.

**Fig. 5:**
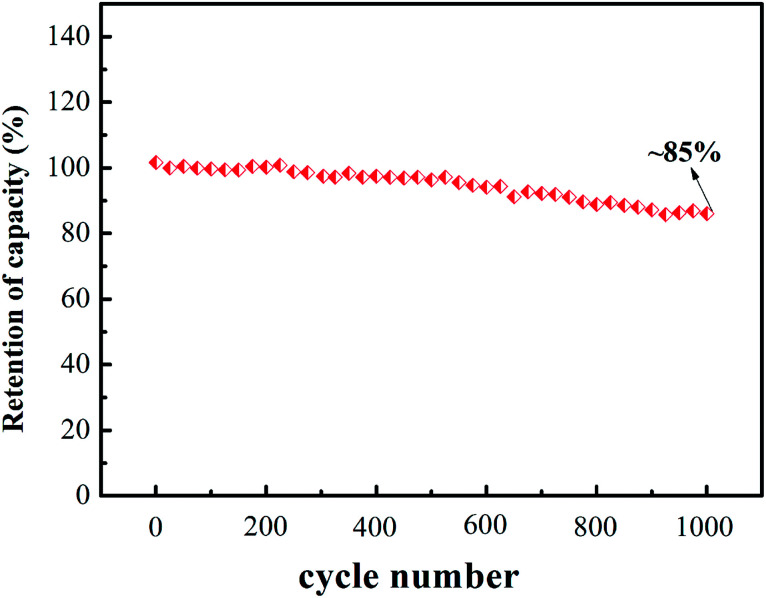
Cycle stability of V2 at 1 A g^−1^.

The cyclic stability of V2 was also measured at 1 A g^−1^ and the analytical results are shown in Fig. 5. We could observe the specific capacitance retention is about 85% after 1000 galvanostatic charging and discharging cycles.

**Fig. 6:**
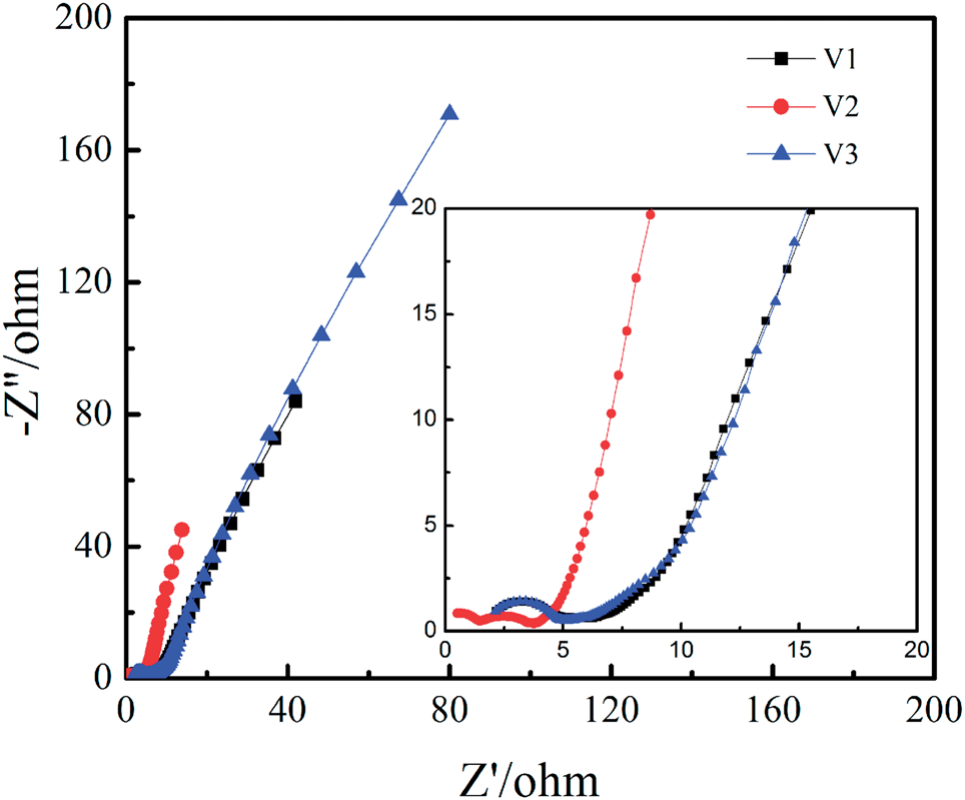
Nyquist plots for V1, V2, and V3.

The Nyquist plots of V1, V2 and V3 can be observed in Fig. 6. It can be seen from the high frequency region that the series equivalent resistance (*R*_s_) of V2 is obviously smaller than V1 and V3. It can be seen in the high frequency region there are two semicircles for V2 and only one semicircle for V1 and V3, implying more redox reaction for V2. The reason for two semicircles in Nyquist plot while four pairs of redox peaks in CV curve is probably the time constants of some redox peaks are similar to each other. It should be noted that the Warburg slope in middle frequency region is steeper for V2, which indicates higher ion mobility and diffusion that is favourable for rate capability and cycling stability.^35^

## 4. Conclusions

In summary, we first synthesized V_2_O_5_ nanowires by a facile capping agent-assisted hydrothermal method with the addition of disodium citrate. When the dosage of disodium citrate is 0.236 g, orthorhombic V_2_O_5_ nanowires were obtained with high crystallinity, high purity and good uniformity. Furthermore, the as-prepared orthorhombic V_2_O_5_ nanowires achieve high capacitance retention, good cycle stability and high specific capacitance of 528.2 F g^−1^ when used as supercapacitor electrode in 0.5 M K_2_SO_4_. In addition, the CV curves with four pairs of peaks reveal that K^+^ ion could intercalation/deintercalation reversibly in the lattice of the orthorhombic V_2_O_5_ nanowires we prepared.

## Conflicts of interest

There are no conflicts to declare.

## Supplementary Material
